# Effects of Phosphorylation on the Activity, Inhibition and Stability of Carbonic Anhydrases

**DOI:** 10.3390/ijms24119275

**Published:** 2023-05-25

**Authors:** Xiaojing Huang, Daniel Winter, Dominic J. Glover, Claudiu T. Supuran, William A. Donald

**Affiliations:** 1 School of Chemistry, University of New South Wales, Sydney, NSW 2052, Australia; 2 School of Biotechnology & Biomolecular Sciences, University of New South Wales, Sydney, NSW 2052, Australia; 3 Neurofarba Department, Sezione di Scienze Farmaceutiche, Universita degli Studi di Firenze, Via Ugo Schiff 6, Sesto Fiorentino, 50019 Florence, Italy

**Keywords:** human carbonic anhydrase I, human carbonic anhydrase II, post-translational modifications, phosphorylation, sulphonamides, esterase activity, carbonic anhydrase inhibition

## Abstract

Carbonic anhydrases (CAs) are a metalloenzyme family that have important roles in cellular processes including pH homeostasis and have been implicated in multiple pathological conditions. Small molecule inhibitors have been developed to target carbonic anhydrases, but the effects of post-translational modifications (PTMs) on the activity and inhibition profiles of these enzymes remain unclear. Here, we investigate the effects of phosphorylation, the most prevalent carbonic anhydrase PTM, on the activities and drug-binding affinities of human CAI and CAII, two heavily modified active isozymes. Using serine to glutamic acid (S > E) mutations to mimic the effect of phosphorylation, we demonstrate that phosphomimics at a single site can significantly increase or decrease the catalytic efficiencies of CAs, depending on both the position of the modification and the CA isoform. We also show that the S > E mutation at Ser50 of hCAII decreases the binding affinities of hCAII with well-characterized sulphonamide inhibitors including by over 800-fold for acetazolamide. Our findings suggest that CA phosphorylation may serve as a regulatory mechanism for enzymatic activity, and affect the binding affinity and specificity of small, drug and drug-like molecules. This work should motivate future studies examining the PTM-modification forms of CAs and their distributions, which should provide insights into CA physiopathological functions and facilitate the development of ‘modform-specific’ carbonic anhydrase inhibitors.

## 1. Introduction

Carbonic anhydrases (CAs) are a ubiquitous metalloenzyme superfamily that catalyse the reversible hydration of carbon dioxide. This process has a crucial role in several physiological functions including pH balance [1], respiration [2], ion transport [3,4] and metabolism [5]. Modulating the activity of carbonic anhydrases can be used to treat multiple pathological conditions including glaucoma [6], epilepsy [7], cancer [1] and neurodegenerative diseases [8]. Thus, there has been significant interest in investigating the structural and biophysical basis of carbonic anhydrase activity and developing selective pharmaceuticals targeting CAs [9], such as sulphonamides for inhibiting CAs in multiple pathological conditions [10]. However, human carbonic anhydrases (hCAs) can be heavily modified by an assortment of post-translational modifications (PTMs) at many different residues [11], and the impact of such modifications on carbonic anhydrase enzymatic activity remains largely unknown.

Information regarding the types and sites of post-translational modifications for many carbonic anhydrase isozymes is scattered across several proteomics databases (i.e., PhosphoSitePlus [12], qPhos [13] and dbPAF [14]). De Simone and co-workers elegantly analysed all PTMs that have been reported in these databases for hCAs [11]. The most commonly occurring PTM for hCAs is phosphorylation, followed by non-enzymatic glycosylation and ubiquitination [11]. Ubiquitination mostly directs protein for degradation [15], while non-enzymatic glycosylation is irreversible as a result of hyperglycaemic conditions, such as diabetes, which can lead to protein cross-linking and loss of function [16]. As a tightly regulated reversible modification, phosphorylation can affect protein activity by altering the protein conformation [17], interactions with binding partners [18] and subcellular localization [19]. Specifically for carbonic anhydrases, recent studies reported an increase in the cytosolic CA activity upon phosphorylation in rainbow trout gills [20] and translocation of phosphorylated luminal CA in *Chlamydomonas reinhardtii* [21]. Despite many documented human CA phosphosites [12,13,14], whether such PTMs significantly alter the biophysical properties of human CAs has not been investigated experimentally. Identifying functional phosphorylation sites in CAs should be beneficial for better understanding the biological roles of CAs and in further developing therapeutic strategies based on carbonic anhydrase inhibition.

There are fifteen known human CAs, including twelve catalytically active isozymes, that are distributed differentially in organs, tissues and biological fluids [22]. Out of the twelve hCAs that are catalytically active, the three cytosolic isozymes hCAI, hCAII and hCAIII have the highest number of phosphorylation sites that have been detected by high-throughput proteomics (15, 14 and 22 respective phosphosites) [11]. These data are consistent with cytosolic proteins being generally over-represented of those that are phosphorylated in vivo [23]. However, the physiological relevance of these sites in terms of hCA enzymatic activity and the specificity of the phosphorylation of folded and intact hCAs has not been confirmed. The sulphonamide-resistant hCAIII is significantly less catalytically active than hCAII by over 100-fold, and has roles in cellular functions beyond its catalytic activity [24]. The catalytically active sites of the active hCAs are highly conserved and consist of a water/hydroxide-bound zinc ion coordinated by three histidine residues deep within a large catalytic cavity that is ~12 Å wide and ~13 Å deep. The cavity is amphiphilic with two sides that consist predominantly of either hydrophilic or hydrophobic residues. The catalytically active zinc hydroxide species can nucleophilically attack the substrate carbon dioxide to form a zinc-bound carbonate that is released to solvent and replaced by a zinc-coordinated water molecule. To regenerate the active zinc-hydroxide in the rate-limiting step of the catalytic cycle, a proton is shuttled from the zinc-bound water molecule to bulk solvent through a network of hydrogen bonds in a process that is mediated by His64 (for the hCAII sequence) [25]. In addition to the extremely high activities for CO_2_ hydration, hCAI and hCAII also act as esterases and phosphatases at much slower turnover rates [26]. The esterase and phosphatase activities of CAs are also reduced by sulphonamide inhibitors, and substrate activation likely occurs through nucleophilic attack by the zinc-hydroxide ion, similar to the enzymatic CO_2_ hydration mechanism [26]. The activity of hCAs for CO_2_ and esterase hydration can both be highly sensitive to mutagenesis and are correlated, although bulkier substrates may potentially be more sensitive to mutagenesis. Thus, the esterase activity of CAs can be used as a proxy for their CO_2_ hydration activity [27,28,29]. To date, the effects of phosphorylation on the enzymatic activity and inhibitor-binding affinities of carbonic anhydrases remain largely unexplored in the literature.

Here, we demonstrate that folded and intact hCAI and hCAII can be phosphorylated by kinases in vitro at a conserved serine residue hCAI^Ser49^ and hCAII^Ser48^. The effects of possible phosphorylation on the activity of these enzymes and their affinities towards sulphonamide inhibitors were studied by recombinantly expressing the phosphomimetic enzymes in which a glutamic acid is used to mimic the size and charge of a phosphoserine. Such S > E phosphomimics can function similarly to the corresponding phosphorylated variants in many conditions [30,31]. A total of thirteen phosphomimics were selected based on known phosphorylation sites (and other factors) and recombinantly expressed. The esterase activities of the phosphomimics were efficiently screened in whole-cell lysates using a microplate-reader-based assay. The catalytic efficiencies of the proteins in the cell lysates correlated well with results obtained for purified hCAs. The position of the phosphomimic site and the identity of the hCA isoform that was modified can strongly affect the activities and inhibition profiles of these enzymes.

## 2. Results and Discussion

### 2.1. Phosphorylation Sites of Interest in hCAI and hCAII

**Figure 1:**
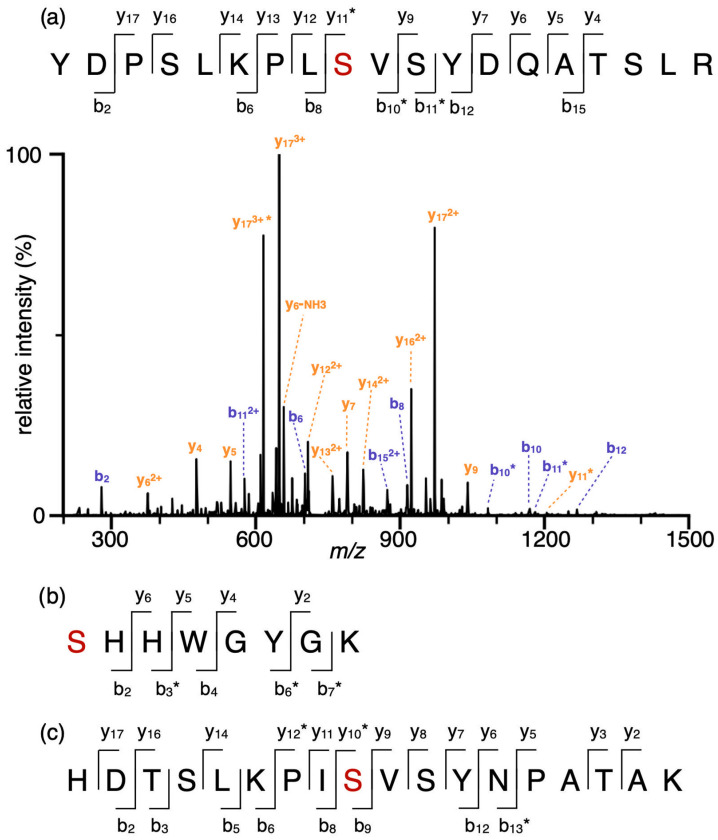
hCAII can be phosphorylated in vitro. After in vitro phosphorylation by PKA and CK2, trypsin-digested peptides were analysed by tandem mass spectrometry. (**a**) The sequence notation of phosphorylated peptide covering Ser48 in hCAII is shown, together with the tandem mass spectrum of 3+ charge state of the indicated peptide. Sequence notations of the peptides covering (**b**) Ser2 in hCAII and (**c**) Ser49 in hCAI. Phosphoserines are labelled with red. Asterisks indicate sequence ions that contain a single phosphorylation site. Sequence ion labels are blue and orange for *b* and *y* ions, respectively.

Although multiple phosphorylation sites have been identified in hCAI and hCAII by high-throughput proteomics experiments [11], none of the sites have been confirmed by alternative approaches. Here, recombinantly expressed unmodified hCAI and hCAII were phosphorylated in vitro using a mixture of protein kinase A (PKA) and casein kinase 2 (CK2) [11]. PKA and CK2 are broad-specificity kinases. Together, they target serine/threonine at both acidic and basic regions of a protein. To identify phosphosites following in vitro phosphorylation, the proteins were isolated in gel, digested with trypsin, and the resulting peptides were analysed by tandem mass spectrometry without any prior enrichment. Phosphoserine modifications were identified at homologous sites, Ser48 (hCAII) and Ser49 (hCAI), with abundances of 8.5% and 0.78% relative to the unmodified site (Figure 1). Additionally, a minor amount (1.6%) of Ser2 phosphorylation was identified for hCAII. The extent of phosphorylation is likely underestimated in these experiments because phosphosites tend to reduce peptide ion signal in positive-mode electrospray ionization [32]. All three residues identified by in vitro phosphorylation were also reported in proteomics databases [12]. These results indicate that human CAs can be phosphorylated as folded and intact proteins, rather than as a result of unspecific kinase activity on unfolded or degraded fragments. Although only a limited number of phosphosites were identified by in vitro phosphorylation, there are hundreds of kinases [33] that can potentially contribute to multiple combinations and permutations of phosphorylation in vivo.

In total, 15 and 14 phosphosites have been reported for hCAI and hCAII, respectively, including phosphorylation at serine, threonine and tyrosine residues [11]. Tyrosine phosphorylation is relatively rare (~2% of global phosphoproteome) [34] and mostly involves receptor kinases for cell signalling pathways [35]. Serine/threonine phosphorylation are common in cells as a regulatory mechanism and can be mimicked by glutamic acid or aspartic acid substitution. In this study, we selected eight serine to glutamic acid (S > E) phosphomimics for hCAII and five phosphomimics for hCAI to investigate (Table 1). Each phosphomimic differs by the position of a single phosphosite, except for hCAII^S48E/S50E^ with two phosphomimetic sites. The sites were selected based on multiple factors, including the in vitro phosphorylation results (i.e., hCAII^S2E^ and hCAII^S48E^; see above), whether the residues are conserved between multiple hCA isoforms, the number of database entries for phosphorylation at a given residue, and structural information.

The eight sites of mutagenesis for hCAII were primarily selected based on the X-ray crystal structure data for hCAII [36]. For example, Ser29 is less than 10 Å from the catalytic zinc centre and Ser172 is ~12 Å from His64, which is a key residue that mediates the proton-shuttle step of the catalytic cycle. Thus, hCAII^S29E^ and hCAII^S172E^ were investigated. Generally, residues that are conserved across multiple isozymes tend to be important functionally and/or structurally [37]. Phosphorylation at Ser50 [38], Ser99 and Ser151 [39,40] of hCAII has been reported multiple times in proteomics databases [3,11,13] and Ser50 and Ser99 are conserved in nine and five different human CA isozymes, respectively (Table 1) [11]. Additionally, Ser151 has a high PhosNet [41] prediction score (0.838) for phosphorylation. Thus, hCAII^S50E^, hCAII^S99E^ and hCAII^S151E^ were also recombinantly expressed. The double phosphomimic hCAII^S48E/S50E^ was selected given the proximity of Ser48 and Ser50, forming a kinase recognition motif (SXSXE) that can potentially be sequentially phosphorylated by acidophilic kinases [42].

**Table 1:** The potential serine phosphorylation sites for hCAII and hCAI were selected based on proteomics data, sequence-based prediction of kinase recognition and catalytically relevant structural considerations.

		Number of Entries for Phosphorylation (PhosphoSitePlus) [12]	Predicted Score *^a^* for Phosphorylation (PhosNet) [41]	Other Factors
hCAII	Ser2	2	n/a	in vitro phosphorylation
	Ser29	3	Unsp. *^b^* (0.998) PKA (0.519)	only serine within 10 Å of the active site
	Ser48	1	Unsp. *^b^* (0.728)	in vitro phosphorylation
	Ser50	2	Unsp. *^b^* (0.565) PKC (0.515)	
	Ser99	3	DNAPK (0.616) PKA (0.522)	
	Ser151	3	Unsp. *^b^* (0.838)	
	Ser172	3	Unsp. *^b^* (0.674)	~12 Å to His64 (relevant to rate-limiting proton shuttle)
hCAI	Ser30	1	Unsp. *^b^* (0.918)	only serine within 10 Å of the active site
	Ser49	1	Unsp. *^b^* (0.887)	in vitro phosphorylation
	Ser51	1	PKC (0.577)	conserved in hCAII
	Ser131	3	Unsp. *^b^* (0.942)	
	Ser137	9	Unsp. *^b^* (0.935)	

*^a^* The higher the PhosNet score, the higher the likelihood that a kinase will phosphorylate a given residue. *^b^* Unspecific: possible phosphorylation by multiple kinases instead of one kinase based on sequence prediction.

Five different hCAI phosphomimics were chosen for recombinant expression (Table 1). Considerably less phosphorylation evidence is reported for hCAI and many selected sites in hCAII do not correspond to serine/threonine residues in hCAI. The phosphomimics hCAI^S30E^ and hCAI^S49E^ were selected owing to the proximity of Ser30 to the catalytic zinc centre [43] and because Ser49 can be phosphorylated in vitro (see above). To investigate the effects of homologous mutants at an additional site, hCAI^S51E^ was also expressed. Mutation at Ser131 of hCAI was chosen because this serine residue is conserved in five isozymes but only phosphorylated for hCAI based on previously reported proteomics data [12]. Therefore, the phosphorylation of Ser131 could potentially have a specific regulatory role for hCAI. Additionally, Ser131 is predicted to be phosphorylated with a high PhosNet score of 0.942 [41]. Finally, Ser137 is a potentially interesting site for phosphorylation based on nine different entries in the database [12] and a high prediction for phosphorylation (PhosNet score 0.935) [41].

### 2.2. Screening Esterase Activity of CAs in Whole-Cell Lysates

**Figure 2:**
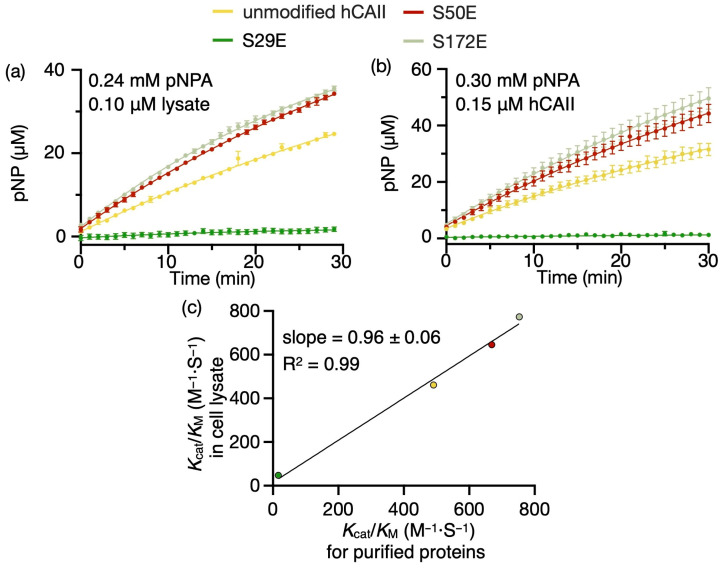
The esterase activity of hCAs can be accurately and efficiently measured in whole-cell lysates. The concentration of the product pNP was obtained as a function of time by measuring the absorbance at 405 nm for (**a**) 0.10 µM unmodified hCAII, hCAII^S29E^, hCAII^S50E^ or hCAII^S172E^ overexpressed in cell lysate incubated with 0.24 mM of the substrate pNPA and (**b**) 0.15 µM of purified unmodified hCAII, hCAII^S29E^, hCAII^S50E^ or hCAII^S172E^ incubated with 0.30 mM pNPA. All reactions were carried out at pH 8.0 and 25 °C. To account for non-enzymatic hydrolysis and latent esterase activity, the absorbance for the reaction mixture was subtracted from that obtained for the same reaction mixture but incubated with a large excess of acetazolamide. Error bars corresponding to ± one standard deviation from replicates (N ≥ 3) are shown. (**c**) The catalytic efficiencies obtained for different hCAII modforms in whole-cell lysates plotted against the catalytic efficiencies obtained for the same enzymes that were purified. The linear regression best-fit line and its slope and correlation coefficient are shown.

To efficiently screen the activity of phosphomimetic hCAs, the enzymatic activities of the CAs were measured in whole-cell lysates without protein purification. In this method, the hydrolysis of *p*-nitrophenyl acetate (pNPA) is monitored as a function of time using the absorbance of the product *p*-nitrophenol (pNP). The concentration of overexpressed enzymes in the cell lysate was normalised using sodium dodecyl-sulfate polyacrylamide gel electrophoresis (SDS-PAGE) and a protein standard (Figure S1). To correct for both the latent esterase activity in the cell lysate and non-enzymatic hydrolysis of pNPA, the absorbance of pNP in the reaction mixture was corrected using the absorbance measurement for the reaction in the presence of a large excess of the well-established carbonic anhydrase inhibitor acetazolamide (Figure S2). In Figure 2a, the concentration of the esterase product is plotted as a function of reaction time for unmodified hCAII and the phosphomimics hCAII^S29E^, hCAII^S50E^ and hCAII^S172E^ in the whole-cell lysate without purification. Interestingly, the enzyme activity depends strongly on the phosphomimetic site. For example, hCAII^S29E^ has essentially no activity in comparison to unmodified hCAII, whereas hCAII^S50E^ and hCAII^S172E^ have higher activities than unmodified hCAII (Figure 2a). The same general trend in the enzyme activity for these four hCAII modforms was also obtained for the purified proteins (Figure 2b and Figure S3). From the initial reaction rates obtained using Equation (1), the catalytic efficiencies (*k*_cat_/*K*_M_) of these enzymes were obtained for the reactions conducted in whole-cell lysates and for purified proteins (Table 1 and Figure 2c). The catalytic efficiencies in the whole-cell lysates correlated strongly with those obtained for the purified proteins (Figure 2c). For example, a linear regression best-fit line of the catalytic efficiencies obtained in the cell lysate vs. purified proteins has a slope of 0.96 ± 0.06 and an intercept of 15 ± 31 that are within the error (95% confidence interval) of the respective values of 1.0 and 0.0 that are expected if there is no deviation between the two approaches. Thus, the activities of the enzymes can be efficiently assessed using whole-cell lysates without protein purification.

### 2.3. Phosphomimic Sites Can Strongly Affect the Esterase Activity of hCAs

**Figure 3:**
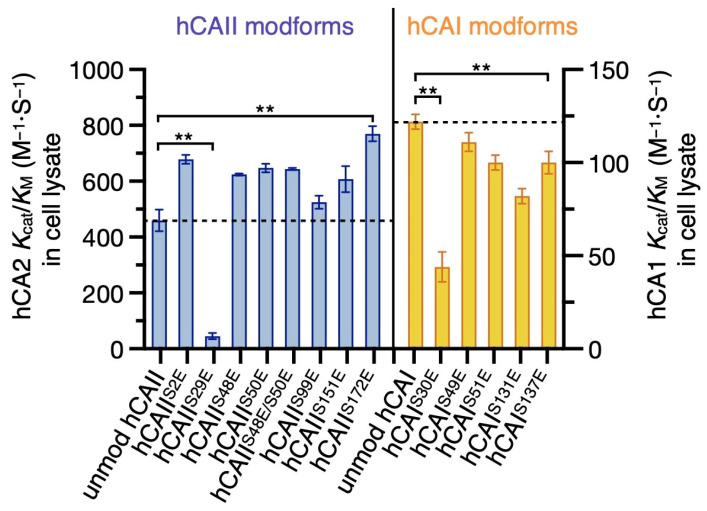
Phosphomimic sites can significantly increase or decrease the catalytic activity of hCAI and hCAII depending on the specific isoform and modification site. The catalytic efficiency (*k*_cat_/*K*_M_) of unmodified and phosphomimetic hCAI and hCAIIs. The efficiencies were obtained from the initial reaction rates. Refer to Figure 2a and Figure S4 for enzyme kinetics measurements and the linear regression fitting to Equation (1) that was used to obtain the initial reaction rates for hCAII and hCAI, respectively. Error bars correspond to the standard deviations from three replicates. Significant changes compared to unmodified hCAI and hCAII are labelled with asterisks.

The esterase activities of all fifteen phosphomimics and unmodified hCAs were measured and their catalytic efficiencies were compared (Figure 3). At the same substrate concentration of 0.24 mM, 1 µM of unmodified hCAI is required to produce a similar signal intensity compared to the 0.1 µM of enzyme required in hCAII measurements (Figure S4 and Figure 2a). The catalytic efficiency of hCAII is 3.8-fold higher than that of hCAI, consistent with a 3.5-fold difference reported previously [26]. Differences in the absolute *k*_cat_/*K*_M_ values obtained in our measurements compared to those reported previously can be attributed to the use of different salt concentrations and buffer pH.

In general, except for hCAII^S29E^, the phosphomimics of hCAII have higher catalytic efficiencies than unmodified hCAII (Figure 3 and Table 2). For example, hCAII^S2E^ and hCAII^S172E^ have 1.5- to 1.7-fold higher catalytic efficiencies than hCAII. The catalytic efficiency of the double phosphomimic hCAII^S48E/S50E^ is higher than the unmodified hCAII, but essentially the same as the corresponding single phosphomimics hCAII^S48E^ and hCAII^S50E^. These data indicate that the double phosphorylation does not have an additive effect in this specific case. For hCAI and hCAII, given there are 10 and 11 reported phosphorylated serine/threonine residues [12], there are 176 and 232 potential states for hCAI and hCAII when considering a maximum of three sites occupied by phosphorylation. Although only a small fraction of these states will be abundant in vivo based on mass spectrometry data for other proteins [44], this efficient approach for screening hCA phosphomimics directly in cell lysates should be useful for investigating specific hCA modforms as proteomics data become available.

In contrast, the phosphomimics of hCAI tend to lower the catalytic efficiencies compared to the unmodified enzyme (Figure 3 and Table 2), suggesting that phosphorylation of two closely related carbonic anhydrase isoforms can result in either enhancing or inhibiting activity. Specifically, the catalytic efficiency of hCAI^S131E^ is 82 ± 4 M^−1^·S^−1^ compared to 122 ± 4 M^−1^·S^−1^ for hCAI. Based on proteomics data, Ser131 is conserved in multiple hCA isozymes but only reported to be phosphorylated in hCAI [12], consistent with an isoform-specific regulation. Taken together with the high number of phosphorylation sites that have been identified in high-throughput proteomics experiments and the esterase activity results, these data are consistent with phosphorylation providing a regulatory mechanism for carbonic anhydrase activity that is isoform-specific.

In addition, S > E mutation at the highly conserved hCAI^Ser30^/hCAII^Ser29^ residue results in 2.7- and 10.5-fold decrease in catalytic activity for hCAI^S30E^ and hCAII^S29E^ compared to the respective unmodified isoforms (Figure 3 and Table 2). The diminished activity of hCAII^S29E^ and hCAI^S30E^ can be attributed to protein unfolding or misfolding upon introduction of a charged residue close to the hydrophobic side of the ligand-binding pocket. The phosphomimic hCAII^S29E^ was only expressed as a soluble protein when using ArcticExpress *E. coli* at 10 °C but not with T7 Express or C43 (DE3) *E. coli*. In ArticExpress cells, a cold-adapted chaperonin is co-expressed to assist folding at low temperatures. In addition, hCAI^S30E^ expresses at a very low yield in the soluble fraction even with the ArcticExpress system, and below detectable levels in T7 Express or C43 (DE3) *E. coli*. Moreover, an S > E mutation at Ser29 in another cytosolic carbonic anhydrase isoform (hCAIII) destabilizes the protein and decreases the melting temperature from 59 °C to 43 °C in differential scanning fluorimetry (Figure S5). Furthermore, native mass spectrometry of hCAII^S29E^ resulted in the formation of protein ions with relatively high charge states (average of 12.7+ compared to 9.9+ for unmodified hCAII; Figure S6), which is consistent with partially unfolded or misfolded proteins [45,46]. Collectively, these results are consistent with phosphorylation at Ser29/30 substantially reducing the stability of hCAI and hCAII and lowering their catalytic activities.

### 2.4. Effects of Phosphomimetic Sites on hCAII Interactions with Sulphonamide Inhibitors

**Figure 4:**
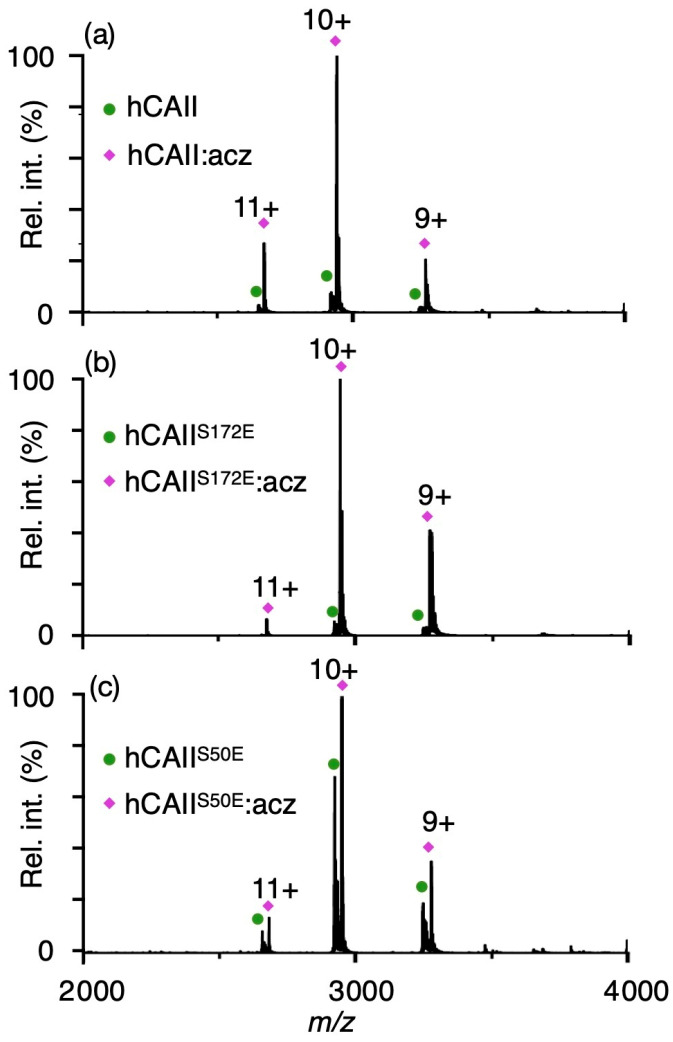
Phosphomimics of hCAII that differ by the position of a single S > E mutation have different binding affinities for the drug acetazolamide (acz). Native mass spectra of solutions containing (**a**) 5:3 µM unmodified hCAII:acz, (**b**) 5:3 µM hCAII^S172E^:acz and (**c**) 5:5 µM hCAII^S50E^:acz in 20 mM ammonium acetate (pH 6.8).

The most well-established drugs and drug-like small molecules that inhibit CAs have a sulphonamide moiety which forms strong interactions with the zinc catalytic centre and thus inhibits the enzymatic activity [47]. The effects of phosphomimetic sites on the interactions of three purified catalytically active hCAII modforms and five FDA-approved hCA-targeting drugs were investigated using native mass spectrometry (Figure 4 and Table 3). Such an approach can be used to measure the binding affinities of sulphonamides [48] and other hCA inhibitors such as carboxylic and sulphonic acids [49] that are in close agreement with those obtained using a well-established stopped-flow kinetic inhibition assay for CO_2_ hydration [50]. Narrow charge state distributions from 9+ to 11+ were formed for unmodified and phosphomimetic hCAII that are consistent with previously reported native mass spectra of hCAII (Figure S7) [48]. In Figure 4a, a native mass spectrum of 5 µM of unmodified hCAII and 3 µM acetazolamide in 20 mM ammonium acetate (pH 6.8) is shown. An abundant series of ions with peaks at *m*/*z* 2673.82, 2940.99 and 3267.66 were detected that correspond to the zinc-bound unmodified hCAII in a complex with acetazolamide (95% relative abundance; Figure 4a). Using the same concentrations, the native MS of hCAII^S172E^ with acetazolamide resulted in ~94% of the CA binding to the inhibitor (Figure 4b), which is essentially the same as that for the unmodified enzyme (~95% of hCAII is bound to acetazolamide; Figure 4a). In stark contrast, only ~61% of hCAII^S50E^ forms a complex with acetazolamide at a higher concentration of sulphonamide (protein/ligand ratio of 5:5 µM; Figure 4c). The dissociation constants (*K*_d_) of hCAII and the five sulphonamides were obtained from these data using methods described previously [48] (Table 3). Although both hCAII^S172E^ and hCAII^S50E^ have higher esterase activities than hCAII, a substantial decrease in the affinity of hCAII^S50E^ for sulphonamides was common for all five compounds (acetazolamide, brinzolamide, dichlorphenamide, ethoxzolamide and indapamide) that were tested (Figure 4 and Figure S8, Table 3). These data indicate that phosphorylation of hCAII at Ser50 can reduce the binding affinities for all five sulphonamide-based inhibitors by 7.4- to 830-fold. For example, unmodified hCAII with the sulphonamide acetazolamide has a measured *K*_d_ that is less than 0.1 µM, but hCAII^S50E^ has an approximately 830-fold higher *K*_d_ value at 83.1 ± 2.4 µM (Table 3). These results indicate that specific phosphomimetic sites can drastically reduce the drug binding affinity of hCAs, and the development of modform-specific inhibitors may be of future interest. Considering the large diversity of CA phosphorylation sites identified by high-throughput proteomics [11], identifying the distribution of naturally occurring CA modforms in vivo may be important for designing both isoform- and modform-specific compounds.

## 3. Materials and Methods

### 3.1. Carbonic Anhydrase Recombinant Expression and Purification

The gene fragments corresponding to unmodified hCAI/hCAII and 13 phosphomimics were designed and purchased from Integrated DNA Technologies. The gene fragments were inserted into the pET-19b backbone via an enzymatic assembly method [51]. All plasmid sequences were confirmed by Sanger sequencing at the Ramaciotti Centre for Genomics (UNSW, Sydney, Australia). The unmodified and phosphomimetic hCAIIs (with exception of hCAII^S29E^) were expressed in C43 (DE3) *E. coli* (New England Biolabs, Ipswich, MA, USA) grown in lysogeny broth (LB) medium at 16 °C overnight with 0.1 mM isopropylthio-β-galactoside (IPTG, Sigma Aldrich, Burlington, MA, USA) and 0.25 mM ZnSO_4_ (Sigma Aldrich, Santa Clara, MA, USA). The unmodified and phosphomimetic hCAIs and hCAII^S29E^ were expressed in ArcticExpress (DE3) (Agilent, Santa Clara, CA, USA) at 10 °C for 2 days with 0.03 mM IPTG and 0.25 mM ZnSO_4_.

Small cell pellets from 5 mL cultures were stored at −20 °C for measuring esterase activity in the cell lysate. To purify hCAII and phosphomimics of hCAII, the cell pellets were lysed by sonication in 50 mM phosphate buffer pH 8.0, 500 mM NaCl and 10 mM imidazole. The lysates were clarified by high-speed centrifugation and the soluble fraction was loaded onto a Ni-NTA column (HisTrap HP, GE Healthcare, Chicago, IL, USA). The His-tagged hCAII was eluted from the column by a stepwise imidazole gradient and dialyzed against 25 mM Tris pH 8.0, 75 mM NaCl and 10 mM β-mercaptoethanol to remove imidazole. The his-tag was cleaved by TEV protease overnight. The tag-free hCAII was collected in the flow-through after reloading onto the Ni-NTA column.

### 3.2. In Vitro Phosphorylation

Recombinantly expressed and purified hCAII, or commercially sourced hCAI (Sigma Aldrich, Burlington, MA, USA), was mixed to a final concentration of 10 µM with 500 enzymatic units each of recombinant protein kinase A (PKA) and casein kinase II (CK2) (New England BioLabs, Ipswich, MA, USA), 1 mM adenosine triphosphate (ATP) and the supplied protein kinase assay buffer to a final volume of 50 µL. The enzymatic phosphorylation reaction was carried out overnight at 30 °C.

The hCAs were isolated using sodium dodecyl sulphate polyacrylamide gel electrophoresis (SDS-PAGE). The band containing hCAs was cut out from the gel and destained by 100 mM ammonium bicarbonate with 50% acetonitrile (MeCN) and dehydrated in 100% MeCN. The gel was reduced by 10 mM dithiothreitol at 55 °C for 30 min and alkylated by 55 mM iodoacetamide for 45 min at room temperature in the dark. The gel was re-equilibrated in 100 mM ammonium bicarbonate and digested with 100 ng trypsin (Promega, Madison, WI, USA) at 37 °C for 16 h. The digested peptides were extracted by 0.1% trifluoroacetic acid (TFA), 0.1% TFA with 60% MeCN and followed by 100% MeCN. The sample was lyophilized by a vacuum concentrator (SpeedVac^@^ Plus SC110A, Savant, Hyannis, MA, USA) and the peptides were reconstituted in 0.1% TFA.

The tryptic peptides were separated online by ultra-high-performance liquid chromatography (Ultimate 3000, Thermo Fisher Scientific, Waltham, MA, USA) and analysed on a high-resolution Orbitrap mass spectrometer (Velos, Thermo Fisher Scientific). An amount of 0.5 µL of sample was injected into an in-house packed C18 column (20 cm × 75 µm). The mobile phases at a flow rate of 0.2 µL/min were composed of solvent A (0.1% formic acid in water) and solvent B (80% MeCN, 0.1% formic acid) with the following gradient profile: 0 to 4 min, 5% B; 36 min, 45% B; 36.5 min, 75% B; 37 min, 75% B; and 37.5 to 48 min, 2% B. The tandem mass spectrometry results were analysed by MaxQuant 2.0.3.1 with variable modifications, oxidation, N-terminal acetylation, S/T phosphorylation and fixed modification carbamidomethylation at cysteine.

### 3.3. Esterase Activity Assay

For measuring the esterase activity in cell lysates, pellets overexpressing hCAs were lysed by protein extraction reagent (BugBuster^®^, Millipore, Burlington, MA, USA). After clarification by high-speed centrifugation, the soluble fraction was diluted ten times in esterase assay buffer (25 mM Tris pH 8.0, 75 mM NaCl, 0.02 mM ZnSO_4_) and left at room temperature for one hour. The hCA concentrations in cell lysates were measured by a comparison to a standard protein (31 kDa, 0.5 µg) on SDS-PAGE. The lysates were then further diluted to 0.2 µM hCAII or 2.0 µM hCAI. For the measurement of the esterase activity of purified proteins, tag-free hCAs were dialyzed into the esterase assay buffer and diluted to 0.3 µM.

The substrate p-nitrophenyl acetate (pNPA) was diluted from stock in ethanol to 0.48 mM or 0.6 mM by esterase assay buffer immediately before the assay. Fifty µL of cell lysates or purified proteins were added into a well (96-well plate, SARSTEDT, Sarstedt, Germany) containing 50 µL of diluted substrate with or without acetazolamide. Triplicate measurements were recorded for each condition. Ten-times-diluted protein extraction reagent was used as a blank at the same reaction time as the hCAII reaction mixture to correct for the delay in measurements. The absorbance at 405 nm was recorded using a plate reader (SPECTROstar Nano, BMG LABTECH, Mornington, VIC, Australia). The absorbance was converted into product concentration by a linear calibration curve generated from 0 to 0.2 mM pNP in the same esterase assay buffer at pH 8.0 and 25 °C (Figure S9). The absorbance measurements were transposed and corrected in GraphPad Prism 9.3.1.

### 3.4. Obtaining Catalytic Efficiencies from Enzyme Kinetics Measurements

To fit the enzyme kinetic data and solve for the initial velocity, an equation from Cao et al. describing a non-linear relationship between product concentration [*P*] and reaction time (*t*) with consideration of product inhibition and/or substrate depletion was modified slightly to incorporate a delay in the measurement time (*a*) [52]. A fitted constant *η* was used to describe the non-linearity in the time course. The inhibitor-corrected enzyme kinetic data were fitted with Equation (1) to solve for the initial velocity (*v*_0_) in GraphPad Prism 9.3.1.

(1) $$\left[P\right]=\frac{v_{0}}{\eta }(1-e^{\eta \left(t+a\right)})$$

The catalytic efficiency (*k*_cat_/*K*_M_) for each variant was calculated based on the Michaelis–Menten Equation (2) under the assumption that *K*_M_ is sufficiently larger than the substrate concentration [*S*] such that *K*_M_ + [*S*] ≃ *K*_M_. Such a condition is valid since the substrate concentration [*S*] used in the esterase assay was 0.3 mM at pH 8.0 while the reported *K*_M_ towards pNPA is 2.92 mM and 15.4 mM at pH 7.8 for hCAII and hCAI, respectively [53].

(2) $$v_{0}=\frac{k_{cat}[E][S]}{K_{M}+[S]}$$

### 3.5. Native Mass Spectrometry and Binding Affinity Measurements

Human CAs were buffer-exchanged into 20 mM ammonium acetate (pH 6.8) by gel filtration columns (Zeba^®^ spin desalting column, Thermo Fisher Scientific). Nanoscale nanoelectrospray ionization emitters with tip inner diameters of ~680 nm were fabricated using a Flaming/Brown-type microcapillary puller (P-97, Sutter Instrument Company, Novato, CA, USA) and coated with a mixture of gold and palladium by a sputter coater (Scancoat six, EDWARDS, Eastbourne, UK) as previously described [48]. Native mass spectra of 5 µM hCA and sulphonamide inhibitors ranging from 3 µM to 10 µM were acquired on a hybrid linear ion trap-Orbitrap mass spectrometer (LTQ Orbitrap XL, Thermo Fisher Scientific) with a voltage of +0.8 to 1.2 kV applied to the nanospray ionization emitters relative to the capillary entrance to the mass spectrometer. Voltages of 160 V and 5 V were applied to the tube lens and capillary (heated to 100 °C), respectively. The integrated abundances of the bound and unbound protein ion charge states (9+, 10+ and 11+) were used to obtain the dissociation constant from the native mass spectra, as reported previously [48].

## 4. Conclusions

Human carbonic anhydrases can be phosphorylated as intact and folded proteins. Potentially functional PTMs that may modulate enzymatic activity can be efficiently and accurately assessed by measuring enzyme activity directly in cell lysates with overexpressed hCA phosphomimics. The catalytic efficiencies obtained using this approach agree well with those obtained for fully purified hCAs. The significant increase or decrease in the catalytic activity of human carbonic anhydrases resulting from S > E phosphomimics are specific to both isoforms and modification sites. For example, phosphomimics at the homologous residue hCAI^S30E^/hCAII^S29E^ almost completely inhibit the esterase activity, while phosphomimics hCAI^S49E^/hCAII^S48E^ enhance the activity of hCAII but inhibit the activity of hCAI.

This research suggests that phosphorylation may be a regulatory mechanism for hCAs, and identifying upstream kinases for hCAs and specific modforms of hCAs that are prevalent in vivo should be beneficial for understanding their pathophysiological functions. Furthermore, carbonic anhydrase phosphomimics can alter enzymatic activity and their inhibition profiles with sulphonamide small-molecule drugs, emphasizing the importance of investigating CA modforms for their pathological relevance and the development of modform-specific therapeutics. Future work will focus on identifying the specific modforms of hCAs in vivo and assessing the impact of the phosphorylation sites of hCAs on the enzyme kinetics of the CO_2_ hydration reaction using a broader scope of inhibitors, such as sulfanilamide, metanilamide and other simple benzene-sulfonamides. Overall, these findings provide new insights into the regulatory mechanisms and functional effects of hCA post-translational modifications, with implications for the development of novel therapeutic approaches targeting this important enzyme family.

## Figures and Tables

**Table 2:** The catalytic efficiency (*k*_cat_/*K*_M_) of human CAs in cell lysate and purified proteins toward p-nitrophenyl acetate.

		Cell Lysate *k*_cat_/*K*_M_ (M^−1^·S^−1^)	Purified Proteins *k*_cat_/*K*_M_ (M^−1^·S^−1^)
hCAII	unmodified	460 ± 38	490 ± 11
	S2E	679 ± 16	
	S29E	45 ± 11	19 ± 13
	S48E	624 ± 4	
	S50E	648 ± 15	670 ± 11
	S48E_S50E	644 ± 4	
	S99E	525 ± 23	
	S151E	607 ± 47	
	S172E	770 ± 27	751 ± 8
hCAI	unmodified	122 ± 4	
	S30E	44 ± 8	
	S49E	111 ± 5	
	S51E	100 ± 4A1	
	S131E	82 ± 4	
	S137E	100 ± 6	

**Table 3:** The binding affinity of sulphonamide drugs depends strongly on the phosphomimic site. Dissociation constants (*K*_d_) obtained by native mass spectrometry for unmodified hCAII and selected catalytically active phosphomimics with five FDA-approved sulphonamide inhibitors.

	Unmodified hCAII	hCAII^S50E^	hCAII^S172E^	
	*K*_d_ (µM)	*K*_d_ (µM)	*K*_d_ (µM)	*K*_i_ (µM) [9]
acetazolamide	<0.1 *^a^* ± 0.1	83.1 ± 2.4	<0.2 *^a^* ± 0.1	0.012
brinzolamide	<2.9 *^a^* ± 1.4	21.5 ± 6.2	<4.2 *^a^* ± 2.5	0.009
dichlorphenamide	<2.8 *^a^* ± 0.4	23.9 ± 0.3	<4.2 *^a^* ± 1.8	0.038
ethoxzolamide	<3.5 *^a^* ± 0.3	84.8 ± 7.5	<2.5 *^a^* ± 0.5	0.008
indapamide	10.0 ± 2.0	102.3 ± 11.3	9.2 ± 2.5	2.52

*^a^* The measured *K*_d_ is higher than the true value due to the saturation of the protein–ligand complex at these concentrations.

## Data Availability

Data is available upon request from the corresponding author.

## Supplementary Materials

The supporting information can be downloaded at https://www.mdpi.com/article/10.3390/ijms24119275/s1.
