# Supplementary material for: Effects of Phosphorylation on the Activity, Inhibition and Stability of Carbonic Anhydrases
Source: Int J Mol Sci. 2023 May 25;24(11):9275. doi: 10.3390/ijms24119275 (PMC10253321; doi:10.3390/ijms24119275)
Supplement: Supplementary file 1 [file ijms-24-09275-s001.zip › ijms-2359058-supplementary.pdf]

Supporting Information

Effects of Phosphorylation on the Activity, Inhibition and Stability of Carbonic Anhydrases

Xiaojing Huang [1], Daniel Winter [1], Dominic J. Glover [2], Claudiu T. Supuran [3] and William A. Donald [1,*]

1 School of Chemistry, University of New South Wales, Sydney, NSW 2052, Australia

2 School of Biotechnology & Biomolecular Sciences, University of New South Wales, Sydney, NSW 2052, Australia

3 Neurofarba Department, Sezione di Scienze Farmaceutiche, Universita degli Studi di Firenze, Via Ugo Schiff 6, Sesto Fiorentino, 50019 Florence, Italy

* Correspondence: w.donald@unsw.edu.au

Table of Contents

Figure S1. SDS-PAGE of cell lysates with overexpressed CAs…………………….....…....S3

Figure S2. Esterase activity of hCAII in cell lysate……………………………..…………S4

Figure S3. Esterase activity of purified hCAII…………………………………...…………..S5

Figure S4. Esterase activity of hCAI in cell lysate…………………………………………...S6

Figure S5. Differential scanning fluorimetry of cytosolic isozyme hCAIII ………………….S7

Figure S6. Native mass spectrum of misfolded/unfolded hCAII^{S29E} …………..………….………S8

Figure S7. Native mass spectra of hCAII, hCAII^{S50E} and hCAII^{S172E} ………………….…………S9

Figure S8. Native mass spectra of hCAII modforms and sulphonamide inhibitors……...….S10

Figure S9. Calibration curve for product p-nitrophenol using 405 nm absorption................S11

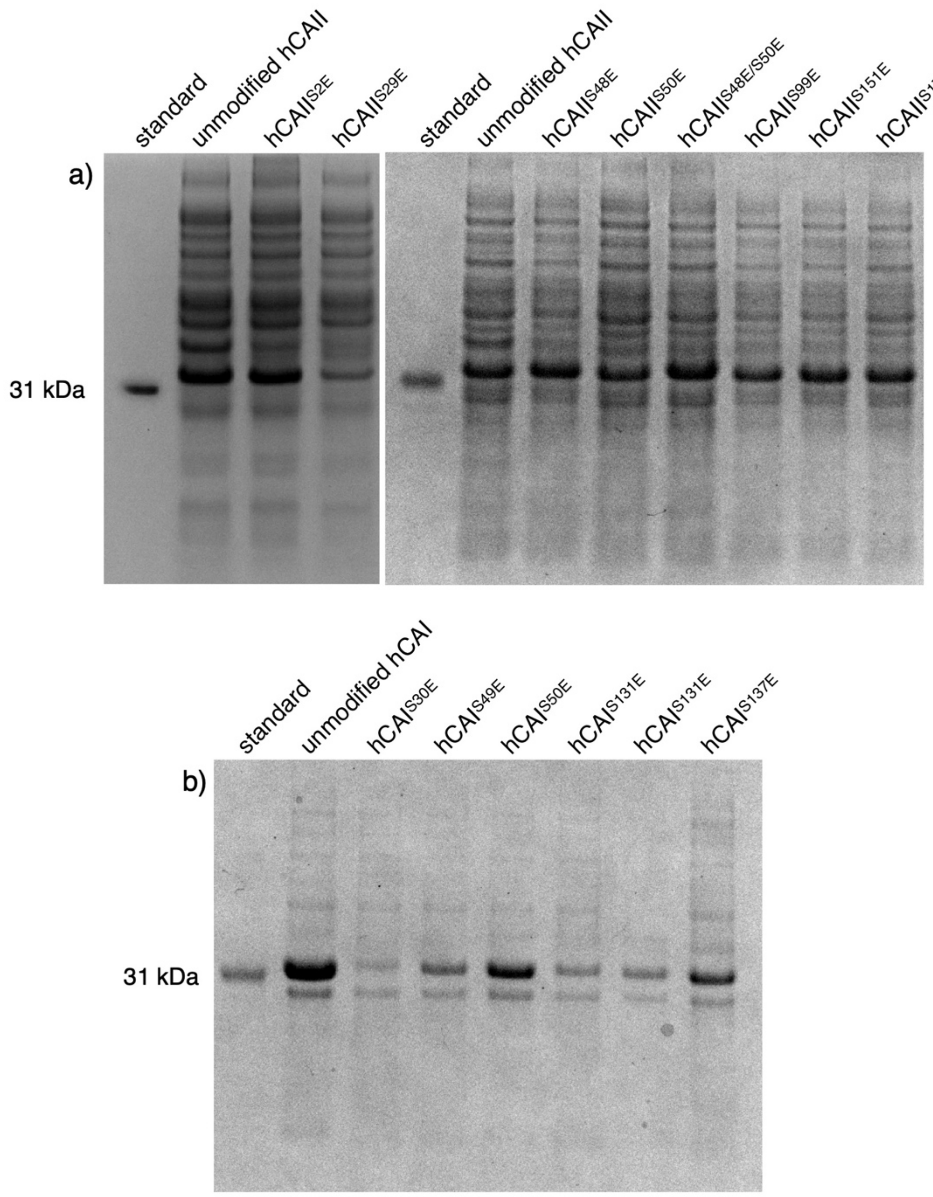

Figure S1. The concentrations of overexpressed unmodified hCAII and hCAI and their phosphomimics in cell lysate were estimated by SDS-PAGE. The overexpressed proteins in bacteria pellets were released by freeze-thaw and soft chemical lysis using BugBuster®. The concentrations of overexpressed (a) hCAII and (b) hCAI were estimated and normalized using a 31 kDa standard protein (STD) at 0.5 μg.

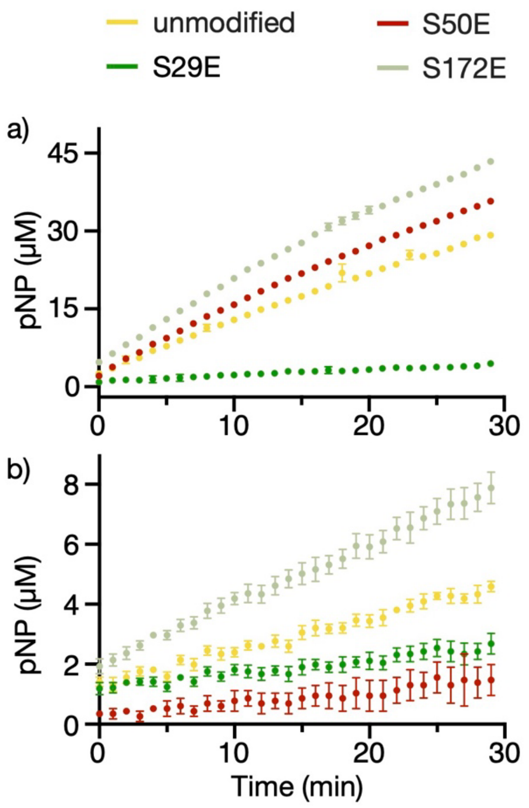

Figure S2. The esterase activity of hCAII modforms in cell lysate measured by tracking the production of reaction product p-nitrophenol (pNP) over time. The concentration of the enzymatic reaction product pNP is plotted as a function of time for (a) 0.1 μM of hCAII modforms overexpressed in cell lysate, and (b) 0.1 μM hCAII modforms in cell lysate with 20 μM acetazolamide. Data was corrected for non-enzymatic hydrolysis by subtracting the reaction blank (i.e. reaction without enzyme added). Refer to Figure 2a for the specific CA enzymatic kinetic data that was corrected using the acetazolamide control data. Error bars correspond to ± one standard deviation from three replicates.

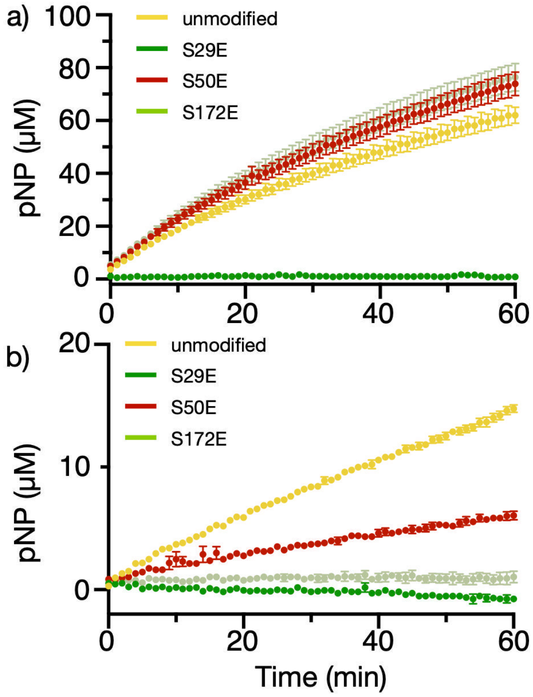

Figure S3. The esterase activity of purified hCA modforms measured by tracking the production of reaction product p-nitrophenol (pNP) over time. The production of pNP concentration was measured using absorbance at 405 nm over time after incubating 0.3 mM of p-nitrophenyl acetate with (a) 0.15 μM unmodified, phosphomimetic hCAII, (b) 0.15 μM unmodified, or phosphomimetic hCAII with 20 μM acetazolamide. Data was corrected using a reaction blank in the absence of enzyme. Refer to figure 2b in main text for specific CA enzyme kinetics corrected with acetazolamide. Error bar corresponds to ± one standard deviation from three replicates.

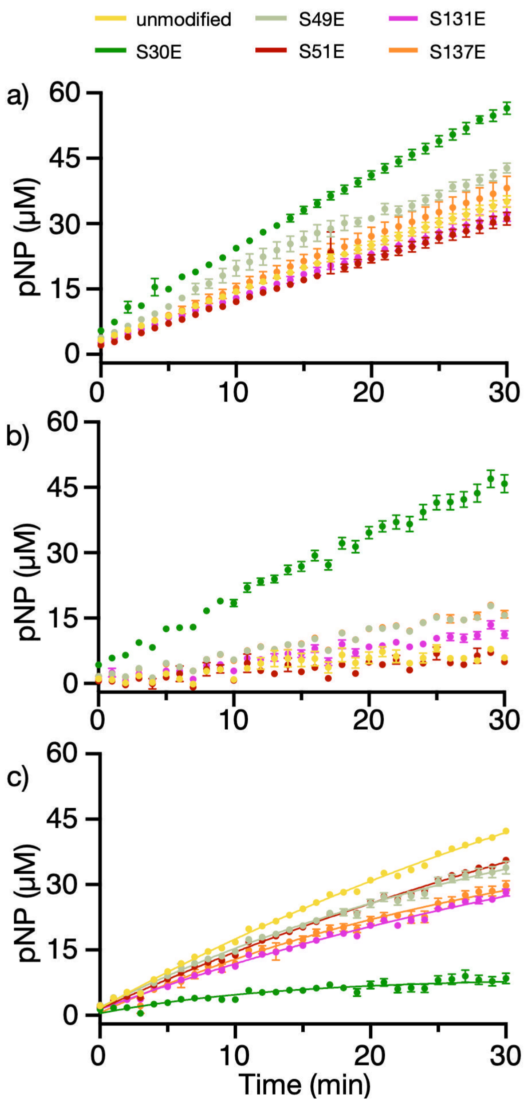

Figure S4. The esterase activity of hCAI modforms in cell lysate measured by tracking the production of reaction product p-nitrophenol (pNP) over time. The product concentration was plotted as a function of time for (a) 1.0 μM hCAI modforms overexpressed in cell lysate, or (b) 1.0 μM hCAI modforms overexpressed in cell lysate with supplement of 50 μM acetazolamide. Data was corrected by blank reaction without enzymes. (c) The CA specific kinetics corrected by acetazolamide control is fitted by equation (1). Error bars correspond to ± one standard deviation from three replicates.

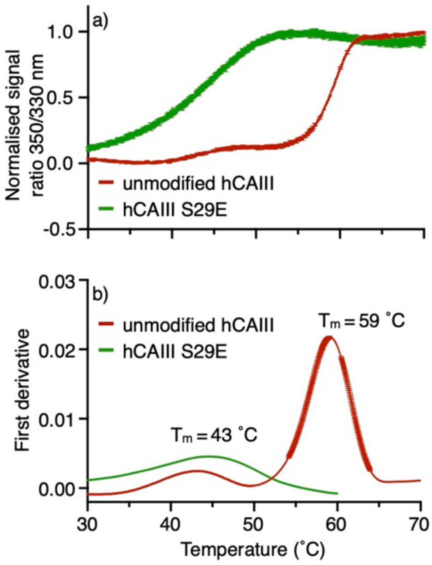

Figure S5. A phosphomimetic site at Ser29 (hCAIII^{S29E}) destabilizes hCAIII. The melting temperature of hCAIII shifts from ~59˚C to ~43˚C for the S>E mutation at residue 29 based on differential scanning fluorimetry experiments (0.2 µM protein, 2 ˚C/min temperature ramp). (a) The ratio of absorbance at 350 nm to 330 nm vs time and (b) the first derivative, which is used to measure the melting temperature that corresponds to unfolding half of the protein.

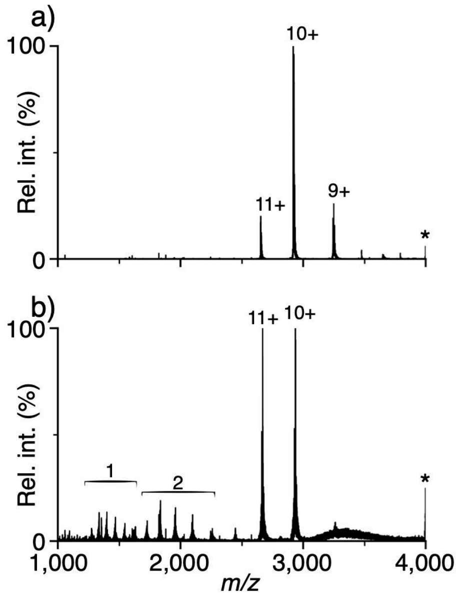

Figure S6. High charge states of hCAII^{S29E} are formed in native mass spectrometry, consistent with low protein stability and partial unfolding in solution compared to unmodified hCAII. Native mass spectra of 5 μM (a) unmodified hCAII and (b) hCAII^{S29E} in 20 mM aqueous ammonium acetate (pH 6.8). The major and folded charge states are labelled from 9+ to 11+. The high charge state distributions in hCAII^{S29E} are labelled by 1 and 2 corresponding to charge states from 18+ to 23+ (1) and 13+ to 17+ (2). Asterisk indicates instrument noise peak.

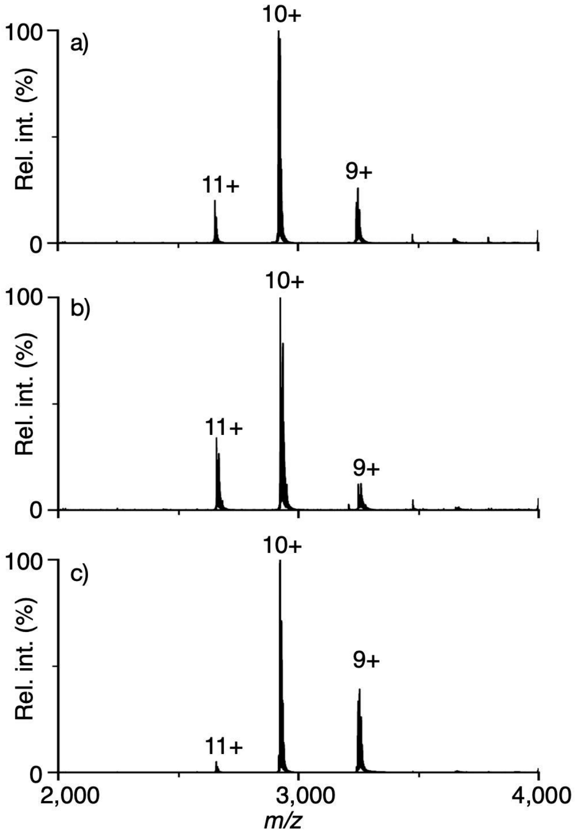

Figure S7. The relatively low, narrow charge states of intact hCAII modforms formed by native mass spectrometry are consistent with these proteins being folded in solution. Native mass spectra of (a) 5 µM unmodified hCAII, (b) 5 µM hCAII^{S50E} and (c) 5 µM hCAII^{S172E}.

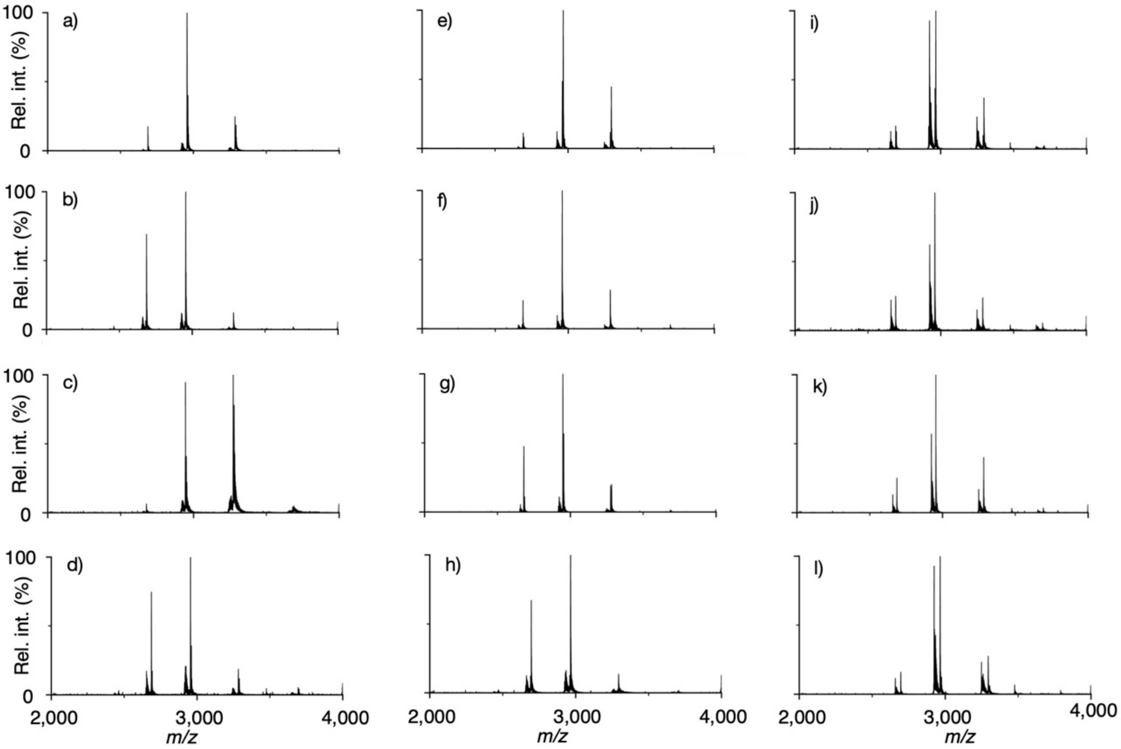

Figure S8. Phosphomimetic sites of hCAII can substantially affect binding affinities with CA-inhibitor drugs. Native mass spectra of 5 μM unmodified hCAII (a-d) and 5 μM hCAII^{S172E} (e-h) with (a, e) 3 μM brinzolamide, (b, f) 3 μM dichlorphenamide, (c, g) 3 μM ethoxzolamide, and (d, h) 5 μM indapamide. Native mass spectra of (i-l) 5 μM hCAII^{S50E} with (i) 5 μM brinzolamide, (j) 5 μM dichlorphenamide, (k) 5 μM ethoxzolamide and (l) 10 μM indapamide.

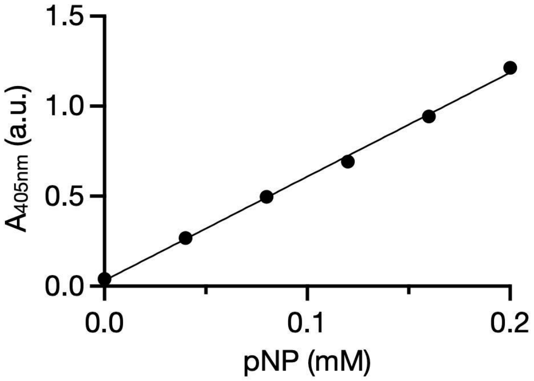

Figure S9. Calibration curve for the enzymatic product p-nitrophenol (pNP). The absorbance at 405 nm as a function of pNP concentration from 0.0 to 0.2 mM, which was measured in the esterase assay buffer at pH 8.0 and 25 ˚C. A simple linear regression best fit line results in $A_{405nm} = 5.774 \times [pNP] - 0.005424$ ($R^2 = 0.997$).
